# Recovery of enzyme activity in biotinidase deficient individuals during early childhood

**DOI:** 10.1002/jimd.12490

**Published:** 2022-03-03

**Authors:** Patrick Forny, Andrea Wicht, Véronique Rüfenacht, Alessio Cremonesi, Johannes Häberle

**Affiliations:** ^1^ Division of Metabolism and Children’s Research Center University Children’s Hospital Zurich, University of Zurich Zurich Switzerland; ^2^ Division of Clinical Biochemistry and Swiss Newborn Screening University Children’s Hospital Zurich, University of Zurich Zurich Switzerland

**Keywords:** biotin therapy, biotinidase deficiency, biotinidase enzyme activity, BTD variants, newborn screening

## Abstract

Deficiency of the biotinidase (BTD) enzyme is an inborn error of biotin metabolism caused by biallelic pathogenic variants in the *BTD* gene. There are two forms, partial and profound BTD deficiency, which both can be successfully treated with pharmacological doses of biotin, justifying the inclusion of this disorder in the newborn screening in numerous countries. We investigated the BTD deficiency cohort (*N* = 87) in our metabolic center, as it was detected upon newborn screening since 2005, and aimed to better understand the long‐term course of BTD enzyme activity and how it may relate to the patients' genetic background. We observed that individuals with partial BTD deficiency display an elevation of BTD enzyme activity with increasing age in 48% of cases—a recovery which allowed adjustment or stop of biotin supplementation in 20% of all individuals. In addition, we were able to recruit 56 patients (64%) for genetic testing, revealing 19 different variants (2 novel), and constituting 22 different genotypes. Genotype–phenotype correlations revealed that the most abundant allele in our cohort p.(Asp444His) was also the most common variant in patients displaying recovery of BTD enzyme activity. Based on our results, we recommend to retest all patients with partial BTD deficiency at the age of 5 years, as this may result in an impact on therapy. Moreover, genetic testing of BTD deficient individuals can allow prediction of the severity of BTD deficiency and of the likelihood of BTD enzyme activity recovery with age.

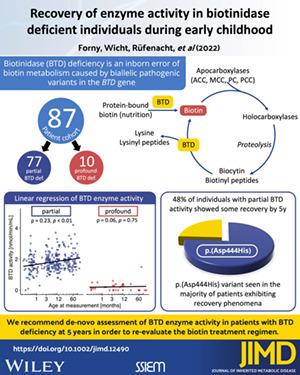

## 1 INTRODUCTION

**FIGURE 1:**
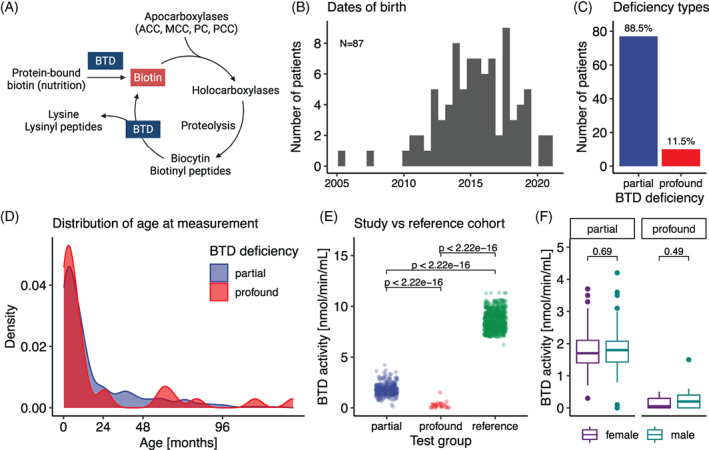
Clinical and enzymatic cohort characterization. (A) Schematic depiction of the biotin cycle, adapted from Zschocke and Hoffmann.^2^ (B) Distribution of dates of birth of each individual in the cohort. (C) Bar chart indicating proportion of individuals with partial and profound BTD deficiency. (D) Distribution of age at biotinidase (BTD) enzyme activity measurements of the full study cohort, subdivided in the two BTD deficiency types. (E) Comparison of all BTD enzyme activity measurements during the study course. (F) Gender comparison per BTD deficiency category. Comparisons indicate *p* values

Biotinidase (BTD) deficiency (OMIM #253260) is an autosomal recessive neurocutaneous inborn error of metabolism, caused by the deficiency of the BTD enzyme (EC 3.5.1.12; encoded by the *BTD* gene), first described in 1983.^1,2^ The assumed homodimer BTD is recycling biotin, a water‐soluble vitamin, by hydrolyzing free biocytin or biotinyl‐peptides originating from the proteolytic degradation of biotin‐dependent carboxylases, where biotin is bound via lysine residues (Figure 1A). Deficiency of this vital function results in four insufficient carboxylation reactions, catalyzed by acetyl‐CoA, propionyl‐CoA, pyruvate, and 3‐methylcrotonyl‐CoA carboxylases, which all require biotin as cofactor.^3^ Individual deficiencies of these enzymes due to biallelic pathogenic variants in their genes are attributed to distinct homonymous inborn errors of metabolism. Lack of the biotin cofactor due to BTD deficiency leads to a severe clinical and distinct biochemical picture, including some features caused by the different deficiencies of the abovementioned holocarboxylase.

Traditionally, the degree of residual BTD enzyme activity defines two related conditions, namely profound BTD deficiency with <10% residual enzyme activity and partial BTD deficiency with 10%–29% residual activity.^4^, ^5^ Untreated patients with profound BTD deficiency have a high risk to develop severe neurological signs and symptoms, including spasticity, seizures, optic atrophy, hearing loss, and cognitive deficits, as well as cutaneous symptoms, such as eczematous skin rash and alopecia.^6^ Patients with untreated partial BTD deficiency can develop mild symptoms of the disorder.^4^ BTD deficiency, both profound and partial, can be successfully treated at low costs with pharmacological doses of orally administered biotin (10 and 5 mg per day for profound and partial BTD deficiency, respectively), leading to the absence of any clinical or biochemical abnormalities.^7^ Hence, BTD deficiency is included in many newborn screening (NBS) programs around the world^8^, ^9^ and in Switzerland has an estimated incidence of 1:50 000 (profound BTD deficiency) and 1:31 000 (partial BTD deficiency) newborns, respectively.

To date, 241 genetic changes are described as pathogenic (*N* = 172) or likely pathogenic (*N* = 69) on the ClinVar database (https://www.ncbi.nlm.nih.gov/clinvar/, accessed August 18, 2021). The most frequently reported pathogenic variant is the missense change p.(Asp444His), which prevents the occurrence of a profound BTD deficiency when present on at least one allele.^10^ Other common pathogenic variants, which are more consistently associated with profound BTD deficiency when present on both alleles, include p.(Cys33Phefs*36), p.(Gln456His), and the *in cis* combination of p.(Ala171Thr) with p.(Asp444His).^11^, ^12^, ^13^

Following a positive NBS result, BTD deficiency is confirmed by genetic means and/or serum enzyme activity measurements. Due to physiologic fluctuations of the measured residual BTD activity, two or three measurements are obtained during infancy as a common practice, enabling reliable categorization into either profound or partial BTD deficiency. Since we observed a relevant increase of BTD enzyme activity in single patients in early childhood, we decided to systematically investigate the natural course of BTD enzyme activity with increasing age in our cohort of patients. We aimed to examine the age‐dependent trajectories of BTD enzyme activity and correlate the findings from enzymatic studies to the genotype of the patients. The results of our study may prove useful in the management of BTD deficiency patients by recommending a repeat BTD enzyme activity test at an additional time point beyond infancy as the result may impact treatment regimens. Furthermore, our results may enable prediction of the severity of BTD deficiency and of a possible recovery based on the underlying genotype.

## 2 MATERIALS AND METHODS

### 2.1 Study cohort

All patients with BTD deficiency treated at the University Children’s Hospital Zurich, Switzerland, from March 2005 until August 2021 were offered participation in the study. Clinical patient information was obtained by retrospective study of patient records including family history, ethnicity, parental consanguinity as well as urinary organic acid analysis, where available. The study was approved by Swiss Ethics (Basec‐No. 2020‐01763).

### 2.2 BTD enzyme activity in dried blood spot

Swiss NBS introduced BTD deficiency screening in 1987. The BTD enzyme activity in dried blood spots (DBS) was determined semi‐quantitatively using different colorimetric or fluorometric assays.^14^, ^15^ Acylcarnitines are not considered for the NBS of BTD deficiency. During the course of the presented study, the kits from PerkinElmer Inc. and Labsystems Diagnostics Oy have been used, which are utilizing as artificial BTD substrates europium‐labeled biotin (GSP Neonatal biotinidase Kit, PerkinElmer) and biotinyl‐6‐aminoquinoline (Neonatal biotinidase kit, Labsystems Diagnostics Oy), respectively. In the former kit, BTD enzyme activity is inversely proportional to the measured fluorescence, while in the latter kit the measured fluorescence is directly proportional to the activity of the BTD enzyme. The cutoff used in the NBS for abnormal values for both assays is <75 U/L. The screening protocol for BTD deficiency is based on a single measurement. If the result is abnormal, a second measurement is performed in technical duplicates in the same DBS card and the mean of the two results is reported. In case BTD enzyme activity is severely decreased (<30 U/L) at this point, the family is directly contacted by the metabolic center to allow prompt medical examination and retesting of BTD enzyme activity in serum. The turnaround time from sample arrival to initial report is about 36 h. All abnormal results between 30 and 74 U/L require a control measurement in a new DBS card, which is normally collected in the first 5 days after the initial report. In case BTD enzyme activity is decreased in the repeat DBS card, the child will be examined and BTD enzyme activity will be measured in serum for a final diagnosis.

### 2.3 BTD enzyme activity in serum

BTD enzyme activity was determined quantitatively by the colorimetric method (reference range: 7.6–10.6 nmol/min/mL), using biotinyl‐4‐amidobenzoic acid as substrate, as previously published.^16^

### 2.4 Molecular genetic analysis of the *BTD* gene

Genomic DNA was isolated from DBS or from peripheral leukocytes using the QIAamp DNA Mini Kit (QIAGEN) and was amplified by polymerase chain reaction, followed by agarose gel electrophoresis and direct Sanger sequencing according to standard protocols using the BigDye Terminator Cycle Sequencing kit version 1.1 and an ABI 3130 genetic analyzer (Applied Biosystems by Life Technologies Europe BV). Raw sequencing data were analyzed using the SeqPatient module of SEQUENCE PILOT software (JSI Medical Systems). The reference sequence of the *BTD* gene used in this study was NM_000060.4, allowing for simplified comparison of variants to previously published data. However, this reference sequence was updated on May 21, 2019 to NM_001370658.1 as the reference standard of the NCBI’s RefSeqGene project, resulting in a shorter BTD enzyme of 523 amino acids compared to the older version comprising 543 residues. Hence, variants using the current reference sequence are shifted by −20 residues (e.g., c.1270G>C, p.(Asp424His)) compared to the old reference (c.1330G>C, p.(Asp444His)). Nomenclature of variants follows the recommendations of the Human Genome Variation Society (http://www.hgvs.org/mutnomen) and was checked using the online software Mutalyzer (https://mutalyzer.nl/name-checker). Detected variants were compared to the ARUP BTD Mutation Database (https://arup.utah.edu/database/BTD/BTD_display.php).

### 2.5 Statistical testing

R software version 4.1.0 was used to design figures and calculate significance levels (by Wilcoxon signed‐rank test, *p* values <0.05 were regarded as significant). To compare BTD enzyme activity measurements obtained at two different time points, the critical difference was calculated, as previously described.^17^ This expression refers to the minimum difference of two values for BTD enzyme activity in a patient, beyond which the change is considered significant.

## 3 RESULTS

### 3.1 Cohort characterization

We set out to characterize the BTD deficiency cohort at the pediatric metabolic center with the largest catchment area in Switzerland, treated during the period from March 2005 to August 2021. A total of 98 individuals were studied, of which all but two were diagnosed by NBS. The two additional patients had migrated to Switzerland after clinical presentation of BTD deficiency (patient ID 81) and upon screening for developmental delay during metabolic work‐up (patient ID 2) (Table 1). Eleven individuals had to be excluded from further analysis since sufficient enzymatic follow‐up data were not yet available (at least ≥3 measurements of BTD activity in serum), including three individuals who showed a residual BTD enzyme activity of >30% upon NBS follow‐up testing. The remaining 87 patients were included in all further analyses.

**TABLE 1:** Overview of study cohort. Dash indicates that molecular genetic testing was not done

	Allele 1		Allele 2			Mean BTD enzyme activity			
Patient ID	Nucleotide change	Amino acid change	Nucleotide change	Amino acid change	BTD deficiency type^a^	Relative (percent of intraday control)	Absolute (nmol/min/mL)	SD^b^ absolute BTD activity	Change of BTD activity with age
1	c.98_104delins TCC	p.(Cys33Phefs*36)	c.968A>G	p.(His323Arg)	Partial	22.7	2.1	0.45	Up
2	c.98_104delins TCC	p.(Cys33Phefs*36)	c.968A>G	p.(His323Arg)	Partial	27.3	2.4	0.55	Up
3	c.98_104delins TCC	p.(Cys33Phefs*36)	c.1330G>C	p.(Asp444His)	Partial	17	1.4	0.75	Up
4	c.98_104delins TCC	p.(Cys33Phefs*36)	c.1330G>C	p.(Asp444His)	Partial	17.8	1.4	0.26	Up
5	c.98_104delins TCC	p.(Cys33Phefs*36)	c.1330G>C	p.(Asp444His)	Partial	18.6	1.6	0.51	Up
6	c.190G>A	p.(Glu64Lys)	c.1330G>C	p.(Asp444His)	Partial	16.3	1.4	0.06	Unaltered
7	c.470G>A	p.(Arg157His)	c.968A>G	p.(His323Arg)	Partial	35	2.8	0.81	Up
8	c.470G>A	p.(Arg157His)	c.1330G>C	p.(Asp444His)	Partial	20.9	2	0.35	Up
9	c.470G>A	p.(Arg157His)	c.1330G>C	p.(Asp444His)	Partial	24.3	1.9	0.46	Up
10	c.470G>A	p.(Arg157His)	c.1330G>C	p.(Asp444His)	Partial	19.4	1.8	0.17	Unaltered
11	c.485C>T	p.(Ala162Val)	c.1330G>C	p.(Asp444His)	Partial	13.9	1.2	0.27	Up
12	c.485C>T	p.(Ala162Val)	c.1330G>C	p.(Asp444His)	Partial	29.7	2.5	0.32	Unaltered
13	c.485C>T	p.(Ala162Val)	c.1330G>C	p.(Asp444His)	Partial	18.3	1.7	0.57	Up
14	c.485C>T	p.(Ala162Val)	c.1330G>C	p.(Asp444His)	Partial	19.3	1.5	0.31	Down
15	c.485C>T	p.(Ala162Val)	c.1330G>C	p.(Asp444His)	Partial	16	1.4	0.45	Up
16	c.485C>T	p.(Ala162Val)	c.1330G>C	p.(Asp444His)	Partial	25.5	2	0.12	Unaltered
17	c.511G>A; c.1330G>C	p.(Ala171Thr); p.(Asp444His)	c.511G>A	p.(Ala171Thr)	Partial	16.3	1.5	0.36	Up
18	c.511G>A; c.1330G>C	p.(Ala171Thr); p.(Asp444His)	c.1330G>C	p.(Asp444His)	Partial	14.8	1.2	0.35	Up
19	c.511G>A; c.1330G>C	p.(Ala171Thr); p.(Asp444His)	c.1330G>C	p.(Asp444His)	Partial	19.5	1.7	0.29	Up
20	c.511G>A; c.1330G>C	p.(Ala171Thr); p.(Asp444His)	c.1330G>C	p.(Asp444His)	Partial	17.2	1.4	0.19	Unaltered
21	c.594_596delCGT	p.(Val199del)	c.1330G>C	p.(Asp444His)	Partial	19.8	1.7	0.27	Up
22	c.625C>T	p.(Arg209Cys)	c.1330G>C	p.(Asp444His)	Partial	35.4	3	0.92	Up
23	c.641A>G	p.(Asn214Ser)	c.1330G>C	p.(Asp444His)	Partial	26.7	2.5	0.64	Down
24	c.641A>G	p.(Asn214Ser)	c.1330G>C	p.(Asp444His)	Partial	28.5	2.4	0.36	Up
25	c.933delT	p.(Ser311Argfs*23)	c.1330G>C	p.(Asp444His)	Partial	20.2	1.8	0.3	Up
26	c.933delT	p.(Ser311Argfs*23)	c.1330G>C	p.(Asp444His)	Partial	18.1	1.6	0.18	Unaltered
27	c.933delT	p.(Ser311Argfs*23)	c.1330G>C	p.(Asp444His)	Partial	22.3	2	0.49	Down
28	c.1267T>C	p.(Cys423Arg)	c.1330G>C	p.(Asp444His)	Partial	23.1	2.2	0.35	Unaltered
29	c.1330G>C	p.(Asp444His)	c.933delT	p.(Ser311Argfs*23)	Partial	20.3	1.7	0.31	Down
30	c.1330G>C	p.(Asp444His)	c.933delT	p.(Ser311Argfs*23)	Partial	11.7	0.9	0.72	Up
31	c.1330G>C	p.(Asp444His)	c.1330G>C	p.(Asp444His)	Partial	29.4	2.5	0.86	Up
32	c.1330G>C	p.(Asp444His)	c.1595C>T	p.(Thr532Met)	Partial	20.2	2.1	1.24	Down
33	c.1330G>C	p.(Asp444His)	c.1612C>T	p.(Arg538Cys)	Partial	20.1	1.8	0.4	Down
34	c.1368A>C	p.(Gln456His)	c.1330G>C	p.(Asp444His)	Partial	19.7	1.6	0.47	Up
35	c.1368A>C	p.(Gln456His)	c.1330G>C	p.(Asp444His)	Partial	26.4	2.2	0.49	Down
36	c.1368A>C	p.(Gln456His)	c.1330G>C	p.(Asp444His)	Partial	19.7	1.9	0.21	Unaltered
37	c.1368A>C	p.(Gln456His)	c.1330G>C	p.(Asp444His)	Partial	20.4	1.8	0.49	Up
38	c.1368A>C	p.(Gln456His)	c.1330G>C	p.(Asp444His)	Partial	19.5	1.7	0.15	Unaltered
39	c.1368A>C	p.(Gln456His)	c.1330G>C	p.(Asp444His)	Partial	20.3	1.8	0.21	Unaltered
40	c.1368A>C	p.(Gln456His)	c.1330G>C	p.(Asp444His)	Partial	19.6	1.8	0.65	Up
41	c.1368A>C	p.(Gln456His)	c.1330G>C	p.(Asp444His)	Partial	23	1.8	0.14	Unaltered
42	c.1368A>C	p.(Gln456His)	c.1330G>C	p.(Asp444His)	Partial	24.7	2.1	0.3	Unaltered
43	c.1368A>C	p.(Gln456His)	c.1330G>C	p.(Asp444His)	Partial	19.6	1.8	0.62	Down
44	c.1368A>C	p.(Gln456His)	c.1330G>C	p.(Asp444His)	Partial	25.9	2.1	0.52	Down
45	c.1368A>C	p.(Gln456His)	c.1330G>C	p.(Asp444His)	Partial	25	2.2	0.26	Unaltered
46	c.1595C>T	p.(Thr532Met)	c.1330G>C	p.(Asp444His)	Partial	17	1.5	0.17	Unaltered
47	c.1595C>T	p.(Thr532Met)	c.1330G>C	p.(Asp444His)	Partial	20.3	2.2	0.96	Up
48	c.1612C>T	p.(Arg538Cys)	c.1330G>C	p.(Asp444His)	Partial	25.8	2	0.54	Down
49	c.1612C>T	p.(Arg538Cys)	c.1330G>C	p.(Asp444His)	Partial	16.3	1.8	0.41	Up
50	c.1612C>T	p.(Arg538Cys)	c.1330G>C	p.(Asp444His)	Partial	19.7	1.6	0.67	Up
51	—	—	—	—	Partial	18.4	1.5	0.2	Unaltered
52	—	—	—	—	Partial	20.4	1.7	0.43	Up
53	—	—	—	—	Partial	20.1	1.7	0.57	Down
54	—	—	—	—	Partial	22.5	1.9	0.4	Up
55	—	—	—	—	Partial	18.7	1.7	0.25	Unaltered
56	—	—	—	—	Partial	23.1	2	0.86	Up
57	—	—	—	—	Partial	20.4	1.8	0.83	Up
58	—	—	—	—	Partial	21.4	1.9	0.69	Down
59	—	—	—	—	Partial	13.3	1.2	0.6	Up
60	—	—	—	—	Partial	15.8	1.3	0.33	Up
61	—	—	—	—	Partial	18.3	1.6	0.2	Unaltered
62	—	—	—	—	Partial	15.7	1.4	0.26	Down
63	—	—	—	—	Partial	17	1.5	0.3	Down
64	—	—	—	—	Partial	22.3	1.9	0.45	Down
65	—	—	—	—	Partial	28	2.3	0.26	Unaltered
66	—	—	—	—	Partial	28.3	2.3	0.55	Up
67	—	—	—	—	Partial	15.4	1.3	0.4	Up
68	—	—	—	—	Partial	23.3	2	0	Unaltered
69	—	—	—	—	Partial	17.7	1.5	0.06	Unaltered
70	—	—	—	—	Partial	14.7	1.2	0.5	Up
71	—	—	—	—	Partial	22.7	1.7	0.17	Unaltered
72	—	—	—	—	Partial	11	0.9	0.1	Unaltered
73	—	—	—	—	Partial	32	2.6	0.54	Up
74	—	—	—	—	Partial	29	2.4	0.88	Up
75	—	—	—	—	Partial	23	1.8	0.31	Up
76	—	—	—	—	Partial	24.5	2	0.2	Unaltered
77	—	—	—	—	Partial	23.2	1.9	0.15	Unaltered
78	c.98_104delins TCC	p.(Cys33Phefs*36)	c.98_104delins TCC	p.(Cys33Phefs*36)	Profound	0	0	0	Unaltered
85	c.98_104delins TCC	p.(Cys33Phefs*36)	c.98_104delins TCC	p.(Cys33Phefs*36)	Profound	1.8	0.2	0.2	Up
79	c.98_104delins TCC	p.(Cys33Phefs*36)	c.469C>T	p.(Arg157Cys)	Profound	3	0.2	0.12	Up
80	c.460‐2A>G	p.?	c.460‐2A>G	p.?	Profound	0	0	0	Unaltered
81	c.460‐2A>G	p.?	c.460‐2A>G	p.?	Profound	0.4	0	0.06	Down
82	c.1368A>C	p.(Gln456His)	c.1368A>C	p.(Gln456His)	Profound	4.1	0.3	0.25	Up
83	c.1409C>T	p.(Thr470Ile)	c.1368A>C	p.(Gln456His)	Profound	5.7	0.5	0.71	Down
84	—	—	—	—	Profound	4.3	0.4	0.1	Down
86	—	—	—	—	Profound	3.5	0.3	0.12	Up
87	—	—	—	—	Profound	1	0.1	0.12	Down

^a^ The category of BTD deficiency reflects the final assessment at the last measurement. Patients with a residual BTD enzyme activity of >29% at the end of the study are still classified as partial deficiency.

^b^ SD, standard deviation.

In line with the study period, the median year of birth in the cohort was 2015 (Figure 1B). The gender distribution was slightly skewed toward male individuals (59.8% males, 52/87) and almost a quarter of patients (24.1%) had at least one other affected sibling in the study cohort. Of the total of 77 families in this study, two were reported as consanguineous, while in 58% of the patients, parents were unable to reliably report on consanguinity status.

### 3.2 Biochemical findings and classification of patients

For the purpose of this study and in line with previous publications and common practice,^4^, ^5^ we defined BTD enzyme deficiency at a cutoff of <30% of residual BTD enzyme activity compared to an intraday control of a mixed reference population, with levels <10% of control defining profound and of 10%–29% defining partial BTD deficiency. Profound BTD deficiency was detected in 10 (11.5%) patients, while partial BTD deficiency was found in 77 (88.5%) patients (Figure 1C). BTD activity was measured up to nine times per individual with an age distribution of these measurements ranging from 7 days to 11.5 years with most of the measurements taking place in the first year of life (Figure 1D). This is in line with the common practice at our center, where each NBS positive patient is followed up three times in the first year of life with enzymatic testing of BTD enzyme activity in serum. Measurements at an older age were obtained under special circumstances (e.g., on parents' request) and for the purpose of this study. In addition to BTD enzyme activity assessments, urinary organic acid profiles were measured in 38 patients at least once, showing normal results in all 59 profiles obtained.

When comparing all measurements of BTD enzyme activity, there was a significant difference between the two types of BTD deficiency regarding the enzyme activity levels, and both types were significantly different from a reference population (reference values resulted from 666 measurements, obtained during the study period) (Figure 1E). The overall mean (each patient’s mean calculated individually and then averaged over all patients) of BTD enzyme activity in the profound deficiency group was 0.21 nmol/min/mL (range 0–0.50), and in the partial deficiency group 1.81 nmol/min/mL (range 0.90–2.96) (Figures 1E and S1A). There was no obvious difference between the normalized distributions of absolute and relative BTD enzyme activity (Figure S1B), and linear regression modeling shows a strong correlation between absolute and relative BTD enzyme activity with a Pearson correlation coefficient of 0.96 (Figure S1C). Hence, for the purpose of this study, we used absolute BTD enzyme activity in all further analyses. There were no significant differences between female and male patients in either group of BTD deficiency (Figure 1F).

### 3.3 BTD enzyme activity recovery with increasing age

**FIGURE 2:**
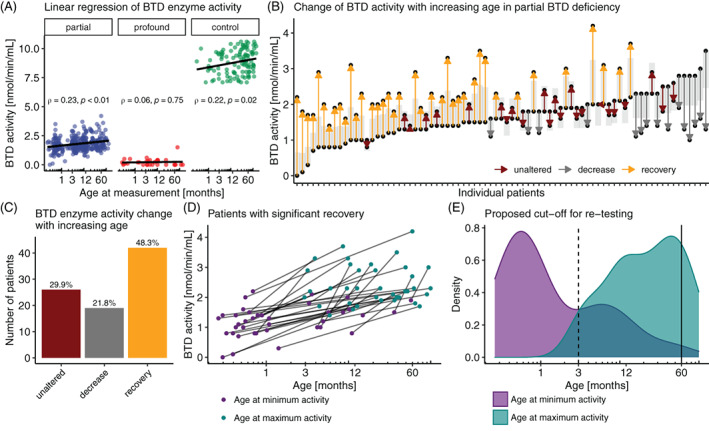
Recovery of biotinidase (BTD) activity with increasing age. (A) BTD enzyme activity data plotted according to age at measurement; linear regressions are calculated per test group. (B) Arrows indicate the chronological relationship between minimal and maximal BTD enzyme activity levels in each individual with partial BTD deficiency; the gray segment indicates the critical difference margin beyond which the change is regarded significant. (C) Bar chart summarizing the changes of BTD enzyme activity with increasing age. (D) Minimal and maximal BTD enzyme activities of the individuals with partial BTD deficiency and significant activity increase with age. (E) Density plot depicting the distribution of ages at minimal and maximal BTD enzyme activity; dotted line indicates the time point at which a majority of patients show recovery of BTD enzyme activity; the solid vertical line indicates the proposed age for retesting BTD enzyme activity in patients with BTD deficiency

In order to define whether there is an age‐dependent change of BTD enzyme activity, we assessed this measure in a longitudinal manner. Linear regression modeling of BTD enzyme revealed a significant correlation to age at measurement in the partial BTD deficiency group but not in the profound category (Figure 2A). While the BTD enzyme activity assay produced stable results over the study period (Figure S2A), we observed a similar development of BTD enzyme activity in a pediatric control group (*N* = 107) with normal BTD enzyme activity (Figure 2A), indicating that BTD enzyme activity generally increases with age during early childhood. To delineate which individuals in the partial BTD deficiency group are driving the phenomenon of BTD enzyme activity recovery, we analyzed each of them by comparing their highest versus their lowest BTD enzyme activity. To define the significance of a change of enzyme activity with increasing age, we calculated the critical difference, based on the variation coefficient (see the Methods section for details) (Figure 2B). Patients with low initial BTD enzyme activity tended to show recovery of BTD enzyme activity with age. Overall, a significant number of patients (48.3%) showed recovery of BTD enzyme activity with age in the partial deficiency group (Figure 2C). A similar proportion of patients fell in the recovery category when assessing the change with increasing age based on the standard deviation of the reference population (Figure S2B). In the group of the patients with significant recovery, the average increase was 1.2 nmol/min/mL (SD = 0.49) (Figure 2D). Already at the age of 3 months, the majority of patients with recovery of BTD enzyme activity changed to their elevated activity, but it is only at the age of 60 months, when 84.2% (32/38) of patients have shifted, suggesting this point in time (5 years of age) as a potential threshold age at which patients might be retested to evaluate whether they show recovery of BTD enzyme activity (Figure 2E).

### 3.4 Reclassification of patients

In the clinical context, changes of BTD deficiency classification were based on the exact percental number obtained for the day‐control‐related residual BTD enzyme activity and not on the assessed significance of changes by the critical difference methodology as applied in this study. If an individual showed a shift from 0%–9% to 10%–29% residual BTD enzyme activity, the classification was changed from profound to partial BTD deficiency. This situation was observed in nine individuals (patient IDs 3, 11, 15, 18, 30, 50, 57, 59, and 70), of which all were indeed found to show BTD enzyme activity recovery according to our calculations based on the critical difference method. Furthermore, if the residual BTD enzyme activity in an individual changed to 30% or more (ascertained in two independent analyses), the biotin supplementation was stopped, and the individual was no longer treated for BTD deficiency. In fact, this constellation was detected in eight individuals according to clinical reports (patient IDs 7, 12, 22, 24, 31, 66, 73, and 74), of which seven showed significant BTD enzyme activity recovery in our study. When two independent measurements from two blood samplings weeks apart had confirmed the BTD recovery, patients were discharged from regular appointments at our metabolic service. These results indicate that for 20% (17/87) of patients the recovery of BTD enzyme activity with age was not only significant in 94% of cases (16/17), but also resulted in a change of management (stop of supplementation of biotin or adaptation of dose).

### 3.5 Identification of pathogenic variants in the *BTD* gene

**FIGURE 3:**
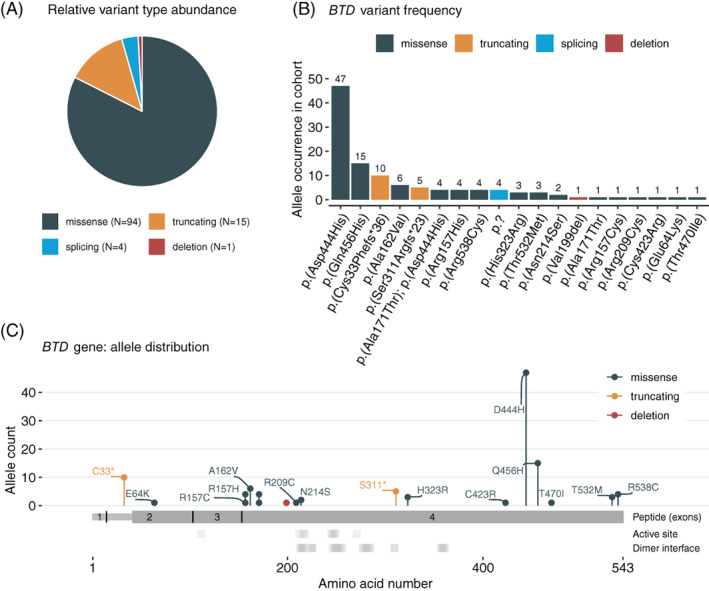
Landscape of *BTD* variants. (A) Pie chart of all variants detected in the cohort and their relative occurrence. (B) Bar chart depicting the frequency of all different variants. (C) Lollipop plot indicating the location and frequency of all variants detected in the cohort. Active site and dimer interface residues are putative based on the reference sequence NP_001357587.1 on the NCBI database, https://www.ncbi.nlm.nih.gov/protein/NP_001357587.1, accessed December 2, 2021); to match the old reference used in our study (and to be in accordance with the nomenclature of variants in the literature) 20 amino acids were added to each residue position. Amino acids 1–41 indicate a signaling peptide

Of the total of 87 patients with enzymatically confirmed BTD deficiency, 57 (65.5%) could be recruited for molecular genetic testing. All individuals were found to carry two disease‐causing variants in the *BTD* gene. In total we found 19 unique variants, of which two were novel genetic changes, namely c.1409C>T (p.(Thr470Ile)) and the splicing variant c.460‐2A>G (Table 2). Missense variants comprised 82.5%, followed by truncating and splicing changes, accounting for 13.2% and 3.5% of variants, respectively (Figure 3A). The detected variants were found to constitute 22 different genotypes. By far the most frequent variant was c.1330G>C encoding for the peptide change p.(Asp444His), constituting 50.0% (47/94) of all missense changes (Figure 3B), which is in line with the high incidence of this variant in literature.^20^ Furthermore, the same missense variant occurred in four patients together with p.(Ala171Thr) on the same allele, as previously described.^21^ Interestingly, p.(Ala171Thr) also occurred once as a single allele, which has only been reported in single cases before.^24^ There were only two unique truncating variants (p.(Cys33Phefs*36) and p.(Ser311Argfs*23)) and only one unique splicing (c.460‐2A>G) and deletion (p.(Val199del)) change, respectively. The splice‐site variant c.460‐2A>G has only been reported once on the ClinVar platform (VCV000619668.1, without clinical evidence) and was found in two siblings of our cohort in a homozygous state. This variant is predicted to affect the splice‐acceptor site of exon 4, leading to alternative splicing. When considering the location of all variants on the BTD polypeptide, no distinct distribution pattern could be identified, except for the two most frequently detected variants in our cohort (p.(Asp444His), p.(Gln456His)), which are located in the region of the mutation cluster at the C‐terminal end of the protein (Figure 3C), as described.^6^ Additionally, we investigated the residues involved in the putative active site and the dimer interface based on structural predictions,^25^ and found two missense variants, both occurring only a single time in the cohort (p.(Arg209Cys), p.(Asn214Ser)), to be located very close to or exactly at one of these residues (Figure 3C), while none of the other variants seemed to interfere with either the residues involved in the active site or dimerization.

**TABLE 2:** Overview of all different variants in the cohort undergoing Sanger sequencing of the *BTD* gene

Nucleotide change	Predicted protein change	Exon/Intron	Allele type	Allele occurrence	Patients	Families	Accession number (ClinVar)	Reference
c.98_104delinsTCC	p.(Cys33Phefs*36)	Exon 2	Truncating	10	8	7	VCV000001895.12	Pomponio et al.^18^
c.190G>A	p.(Glu64Lys)	Exon 2	Missense	1	1	1	RCV000021896.1	Wolf et al.^19^
c.460‐2A>G	p.(?)	Intron 3	Splicing	4	2	1	VCV000619668.1	This study
c.469C>T	p.(Arg157Cys)	Exon 4	Missense	1	1	1	VCV000025012.4	Mühl et al.^20^
c.470G>A	p.(Arg157His)	Exon 4	Missense	4	4	4	VCV000038290.9	Pomponio et al.^18^
c.485C>T	p.(Ala162Val)	Exon 4	Missense	6	6	4	VCV000025014.2	Norrgard et al.^21^
c.511G>A	p.(Ala171Thr)	Exon 4	Missense	1	1	1	VCV000038298.22	Norrgard et al.^12^
c.511G>A; c.1330G>C	p.(Ala171Thr); p.(Asp444His)	Exon 4; Exon 4	Missense; Missense	4	4	4	VCV000025016.5	Norrgard et al.^12^
c.594_596delCGT	p.(Val199del)	Exon 4	Deletion	1	1	1	VCV000587749.1	Pomponio et al.^18^
c.625C>T	p.(Arg209Cys)	Exon 4	Missense	1	1	1	VCV000458809.6	Procter et al.^22^
c.641A>G	p.(Asn214Ser)	Exon 4	Missense	2	2	2	VCV000038579.4	Wolf et al.^23^
c.933delT	p.(Ser311Argfs*23)	Exon 4	Truncating	5	5	4	VCV000025052.11	Pomponio et al.^18^
c.968A>G	p.(His323Arg)	Exon 4	Missense	3	3	2	VCV000038278.9	Swango et al.^10^
c.1267T>C	p.(Cys423Arg)	Exon 4	Missense	1	1	1	VCV000025073.4	Pomponio et al.^18^
c.1330G>C	p.(Asp444His)	Exon 4	Missense	47	46	42	VCV000001900.29	Swango et al.^10^
c.1368A>C	p.(Gln456His)	Exon 4	Missense	15	14	13	VCV000001902.30	Swango et al.^10^
c.1409C>T	p.(Thr470Ile)	Exon 4	Missense	1	1	1	n/a	This study
c.1595C>T	p.(Thr532Met)	Exon 4	Missense	3	3	3	VCV000001897.15	Swango et al.^10^
c.1612C>T	p.(Arg538Cys)	Exon 4	Missense	4	4	4	VCV000001898.11	Pomponio et al.^18^

### 3.6 Functional impact of variants

Since recovery of BTD enzyme activity might be driven by the genetic background of individuals, we investigated the genotype–phenotype relationship by correlating variant classes and certain specific variants to BTD mean activity and change of enzyme activity with age.

**FIGURE 4:**
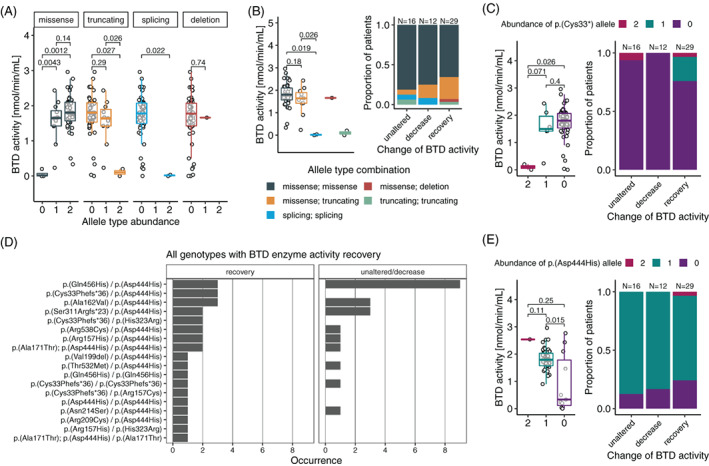
Functional impact of variants. (A) Comparisons of variant classes and their abundance, and (B) different allele combinations with regards to biotinidase (BTD) enzyme activity. (C) Bar chart of all allele combinations which showed BTD enzyme activity recovery in at least one patient. (D + E) Boxplots and proportional bar plots illustrating the impact of a specific mutant allele on BTD enzyme activity and the alteration of said activity with increasing age. Comparisons indicate *p* values

Analysis of the different variant types in relation to mean BTD enzyme activity per individual showed that the absence of a missense allele led to a lower BTD enzyme activity compared to the presence of one or two alleles of this variant type (Figure 4A). A range from low to high residual BTD enzyme activity was detected in patients harboring two missense alleles, as expected from the different effects a specific amino acid exchange might exert on enzyme function and/or stability. An increased presence of truncating alleles showed a nonsignificant tendency toward lower BTD enzyme activity (Figure 4A). The homozygous splicing variant c.460‐2A>G, detected in two patients, led to profound BTD deficiency with virtually absent residual BTD enzyme activity (Figure 4A).

Further examination of the concrete allele combinations, as determined in each patient, and their contribution to the impairment of BTD enzyme activity revealed that missense/missense and missense/truncating combinations occur to be milder than the two abovementioned patients harboring homozygous splicing variants (Figure 4B). When assessing the relationship of each allelic combination and the long‐term development of BTD enzyme activity with increasing age, the missense/missense pattern was present in all three categories of unaltered, decreased and recovery of BTD enzyme activity (Figure 4B). To our surprise, there was an increased proportion of missense/truncating combinations in the category of BTD enzyme activity recovery (Figure 4B), suggesting that one or both of the detected truncating variants may allow the expression of a mutant protein based on the genetic information of the second allele leading to a recovery effect (Figure 2A).

To investigate which of the two truncating variants participates consistently in allele combinations derived from individuals with BTD enzyme activity recovery with age, we inspected all genotypes in the recovery group individually. While the p.(Ser311Argfs*23) variant occurred in all categories (recovery, unaltered, decreased BTD enzyme activity), the other truncating variant p.(Cys33Phefs*36) was only found in patients of the recovery category in combination with a missense allele (Figure 4C). Closer review of this variant revealed that there was no difference in mean BTD enzyme activity when absent or present in the heterozygous state, and that the latter state was clearly enriched in the BTD recovery category (Figures 4D and S3).

In addition, we investigated the most frequently identified allele in the cohort p.(Asp444His) and its impact on BTD enzyme activity and alteration with increasing age. While there was a significant impact of the presence of this specific variant on one allele, leading to a higher mean BTD enzyme activity, as expected from literature,^10^ there was no consistent effect on BTD enzyme activity recovery with age (Figure 4E). However, for all 29 individuals for which we had genetic information and who were assigned to the recovery category, p.(Asp444His) was found in 22 of them (75.9%, 22/29), indicating a relevant contribution of this variant to BTD enzyme recovery (Table 1). Despite the high abundance of the p.(Asp444His) allele, only two individuals (patient ID 31 and one patient not included in the analysis) were harboring this variant in a homozygous state. The first patient showed BTD enzyme activity recovery and treatment with biotin supplementation was stopped at just over 3 years of age. In the second individual, the residual BTD enzyme activity was already at 57% of intraday control at the first follow‐up test following NBS; hence, no further BTD enzyme activity testing has been performed and the patient was excluded from our study cohort. These high residual BTD enzyme activities as observed in the two individuals homozygous for p.(Asp44His) are in line with reports from literature.^10^ To further understand the impact of the genetic background, we analyzed the trajectories of BTD enzyme activity and the categories of BTD enzyme activity change with age in the eight siblings of our cohort, which revealed mostly a consistent course of BTD enzyme activity over time and coherent BTD change categories in five out of eight cases (Figure S4).

## 4 DISCUSSION

BTD deficiency has become a target disease in many NBS programs based on the excellent results of early start of therapy in affected individuals. Treatment decisions are usually made according to the level of the residual BTD enzymatic activity. When we identified single patients with a recovery of their residual BTD activity beyond the threshold of therapeutic necessity when tested during early childhood, we set out to characterize the BTD deficiency cohort in our metabolic center since 2005. We found that partially BTD deficient individuals quite frequently show an increase of their enzyme activity until early childhood and that this recovery can at least partially be attributed to the genetic background.

Without indication of a clear mechanism, there are examples in literature of enzymes whose activities are diminishing with age, including drug‐detoxifying enzymes in rats^26^ and lactase in humans, which shows declining activity in most human populations during mid‐childhood that is thought to be of evolutionary benefit as it promotes weaning.^27^, ^28^ On the other hand, sphingomyelinase and ceramidase, two enzymes involved in sphingolipid metabolism, showed increasing enzyme activities in aging rats.^29^ Hence, change of enzymatic activity with age is a phenomenon which has been observed before. In our patient and control cohorts, individuals with residual or normal BTD enzyme activity showed an age‐dependent increase in BTD enzymatic activity, when assessed during the time span from the neonatal to the young adolescent period. We have shown that this phenomenon impacts the treatment of patients, who may be reclassified from profound to partial BTD deficiency with resulting change in the recommended dose of biotin or even achieve a residual BTD enzyme activity above 30% of control, resulting in a halt of biotin supplementation.

However, the mechanism of this BTD enzyme activity recovery remains unexplained. By investigating the genetic landscape of *BTD* gene variants in our cohort, we were able to partly predict the residual BTD enzyme activity and the potential for BTD enzyme activity recovery with age. We identified one truncating variant in our cohort, p.(Cys33Phefs*36), which was found together with a missense change (p.Asp444His, p.His323Arg, or p.Arg157Cys, respectively) on the other allele in six patients, who all belonged to the recovery category. We speculate that this truncating variant leads to effective nonsense mediated decay (NMD) and hence complete loss of the truncated protein, resulting in a subsequent enzyme dimerization from solely the second allele expressing the missense variant. The resulting amount of BTD protein will accordingly be substantially lower than in the wildtype situation, but the available BTD protein may still be partly functional explaining the partial BTD phenotype. A similar situation should in principle exist also for genotypes carrying the other truncating variant in our cohort, p.Ser311Argfs*23, but this mutation likely leads to much less efficient NMD since there is no intronic sequence downstream of the exon containing the premature stop of translation,^30^ and hence aberrant BTD monomers may result that subsequently interfere with proper dimerization.

Finally, it has been shown that the molecule biotin directly affects the expression of various genes, including biotin‐dependent carboxylases as well as holocarboxylase synthetase (HCS), by influencing intracellular concentrations of the second messenger cGMP.^31^, ^32^ While in vitro studies have shown that reduced biotin availability in BTD deficiency leads to reduced expression of HCS,^33^ the reverse case (increased expression of HCS and potentially BTD under high abundance of biotin) has not been demonstrated yet. Hence, in BTD deficiency treated with pharmacological doses of biotin, a potential mechanism involving high levels of biotin which may act on gene transcription remains to be examined. A next step to better understand this relationship may include the assessment of BTD enzyme activity in patients whose treatment has been stopped upon BTD recovery.

In conclusion, we describe that BTD enzyme activity undergoes a recovery with increasing age, impacting treatment regimens for patients with BTD deficiency. In three quarters of cases, this recovery was associated with the p.(Asp444His) variant on at least one allele, rendering this allele a clear driver of BTD recovery. Based on our findings, we recommend a de novo assessment of BTD enzyme activity in patients with BTD deficiency at the age of 5 years in order to reevaluate the biotin treatment regimen. When biotin supplementation can be stopped based on BTD activities exceeding 30%, occurrence of symptoms is unlikely and not described in literature. We regard patients with “recovered” BTD activities as different from those with partial BTD deficiency, in whom in single cases skin and neurological symptoms were reported after discontinuation of biotin treatment.^8^ Accordingly, we inform the families upon discharge from our specialized metabolic service about the necessity to consider the possibility of biotin deficiency if symptoms such as skin rash, ataxia, hearing, or visual problems would occur. To confirm our novel observation of BTD enzyme activity increase with age, we would welcome if other centers would add the additional BTD enzyme activity measurement to their regular monitoring scheme for this condition.

## CONFLICTS OF INTEREST

The authors have no potential conflict of interest to declare.

## DATA AVAILABILITY

My manuscript has data included as electronic Supplementary Material.

## ETHICS STATEMENT

All procedures were in accordance with the ethical standards of the Helsinki Declaration of 1975, as revised in 2013. As stated in the methods section, patient information and material were obtained and used under the ethics approval no. KEK‐2020‐01763, granted by the Ethics Committee of the Canton of Zurich, Switzerland.

## Supporting information


**Supplementary Figure S1** (a) Box plots of BTD enzyme activity representing all individuals in the cohort. (b) Comparison of the distributions of scaled data for absolute and relative BTD enzyme activity. (c) Linear regression (indicated by the blue line) of absolute and relative BTD enzyme activity; rho and p values are calculated based on the Pearson correlation model.
**Supplementary Figure S2** (a) Chronological depiction of BTD enzyme activity levels of all values obtained in the cohort. (b) Proportions of categories of BTD enzyme activity change with increasing age, depending on two different calculations (for details see methods).
**Supplementary Figure S3** Bar plot indicating the abundance in the categories of BTD enzyme activity change with increasing age for all allele combinations observed in the cohort.
**Supplementary Figure S4** Comparisons of sibling pairs. (a) Course of BTD enzyme activity at different measurement time points. (b) Category of BTD enzyme activity alteration with increasing age
