# Supplementary material for: Recovery of enzyme activity in biotinidase deficient individuals during early childhood
Source: J Inherit Metab Dis. 2022 Mar 3;45(3):605–20. doi: 10.1002/jimd.12490 (PMC9310736; doi:10.1002/jimd.12490)
Supplement: Supplementary file 1 — Supplementary Figure S1 (a) Box plots of BTD enzyme activity representing all individuals in the cohort. (b) Comparison of the distributions of scaled data for absolute and relative BTD enzyme activity. (c) Linear regression (indicated by the blue line) of absolute and relative BTD enzyme activity; rho and p values are calculated based on the Pearson correlation model. Supplementary Figure S2 (a) Chronological depiction of BTD enzyme activity levels of all values obtained in the cohort. (b) Proportions of categories of BTD enzyme activity change with increasing age, depending on two different calculations (for details see methods). Supplementary Figure S3 Bar plot indicating the abundance in the categories of BTD enzyme activity change with increasing age for all allele combinations observed in the cohort. Supplementary Figure S4 Comparisons of sibling pairs. (a) Course of BTD enzyme activity at different measurement time points. (b) Category of BTD enzyme activity alteration with increasing age [file JIMD-45-605-s001.pdf]

Recovery of enzyme activity in biotinidase deficient individuals during early childhood

Forny *et al.*

Supplementary Information

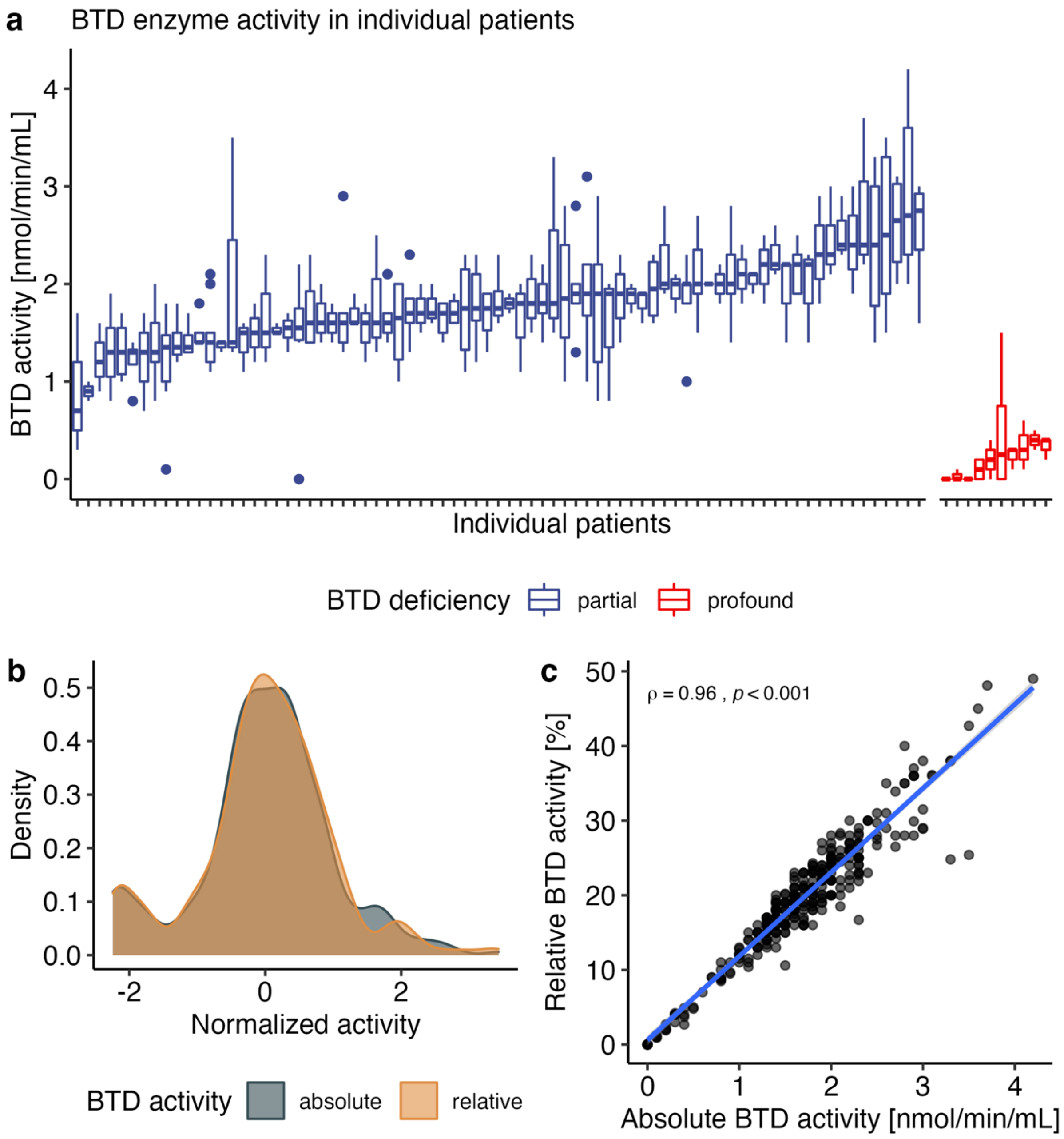

Supplementary Figure 1. (**a**) Box plots of BTD enzyme activity representing all individuals in the cohort. (**b**) Comparison of the distributions of scaled data for absolute and relative BTD enzyme activity. (**c**) Linear regression (indicated by the blue line) of absolute and relative BTD enzyme activity; *rho* and *p* values are calculated based on the Pearson correlation model.

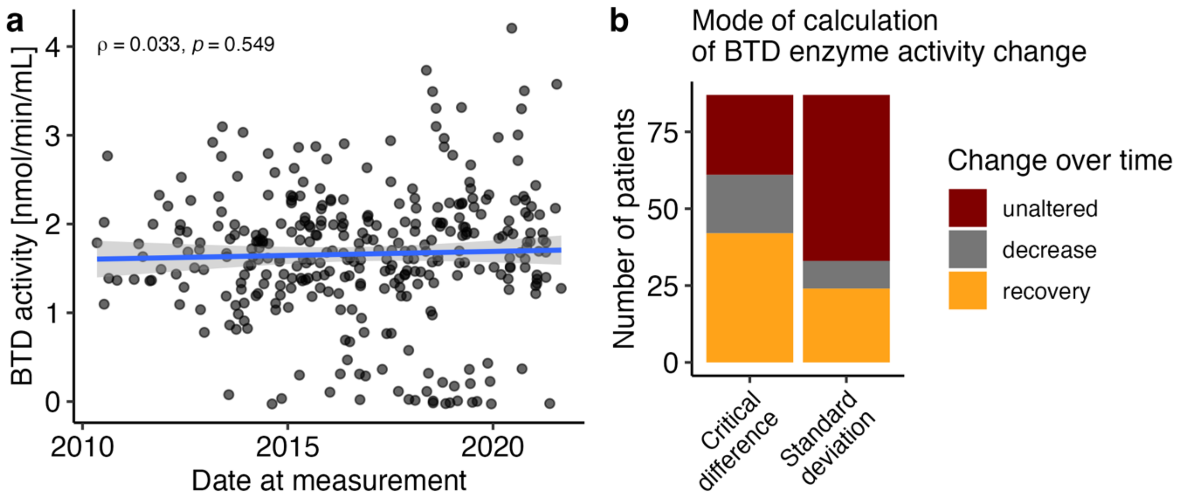

Supplementary Figure 2. (**a**) Chronological depiction of BTD enzyme activity levels of all values obtained in the cohort. (**b**) Proportions of categories of BTD enzyme activity change with increasing age, depending on two different calculations (for details see methods).

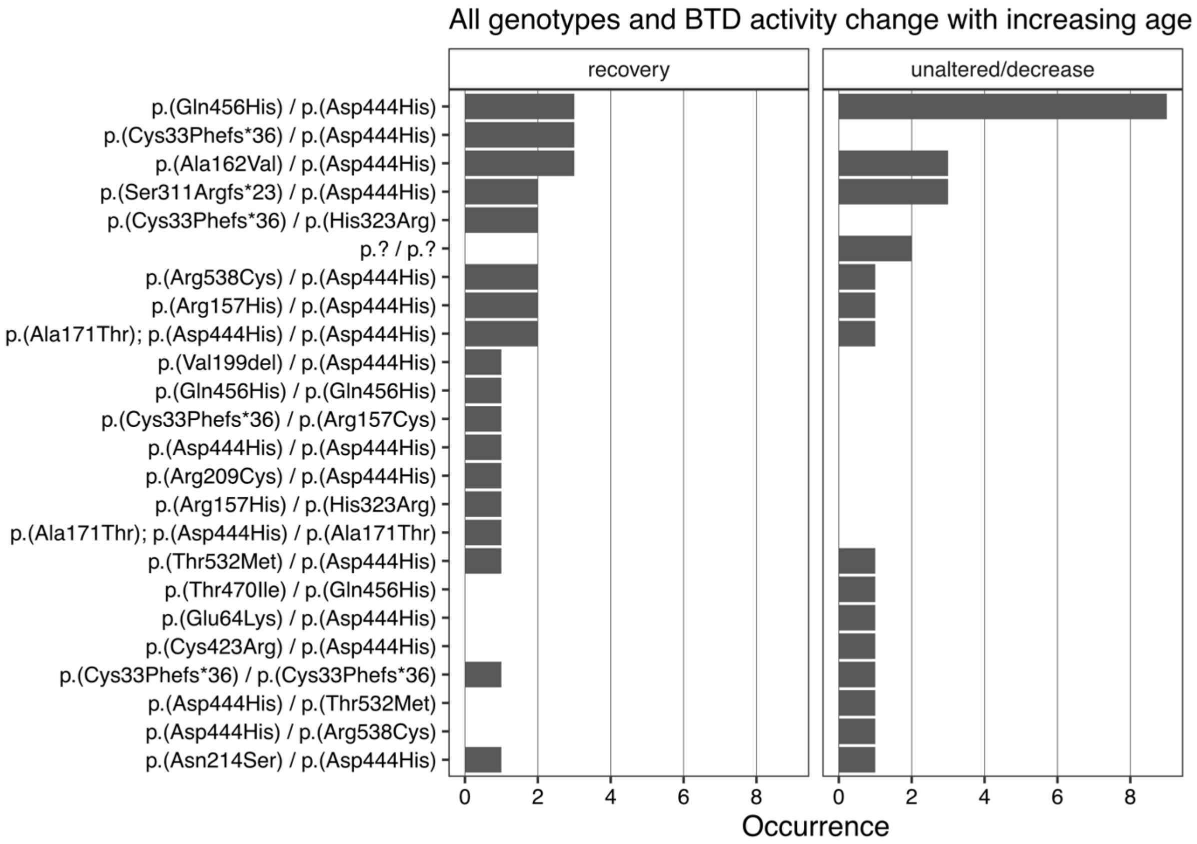

Supplementary Figure 3. Bar plot indicating the abundance in the categories of BTD enzyme activity change with increasing age for all allele combinations observed in the cohort.

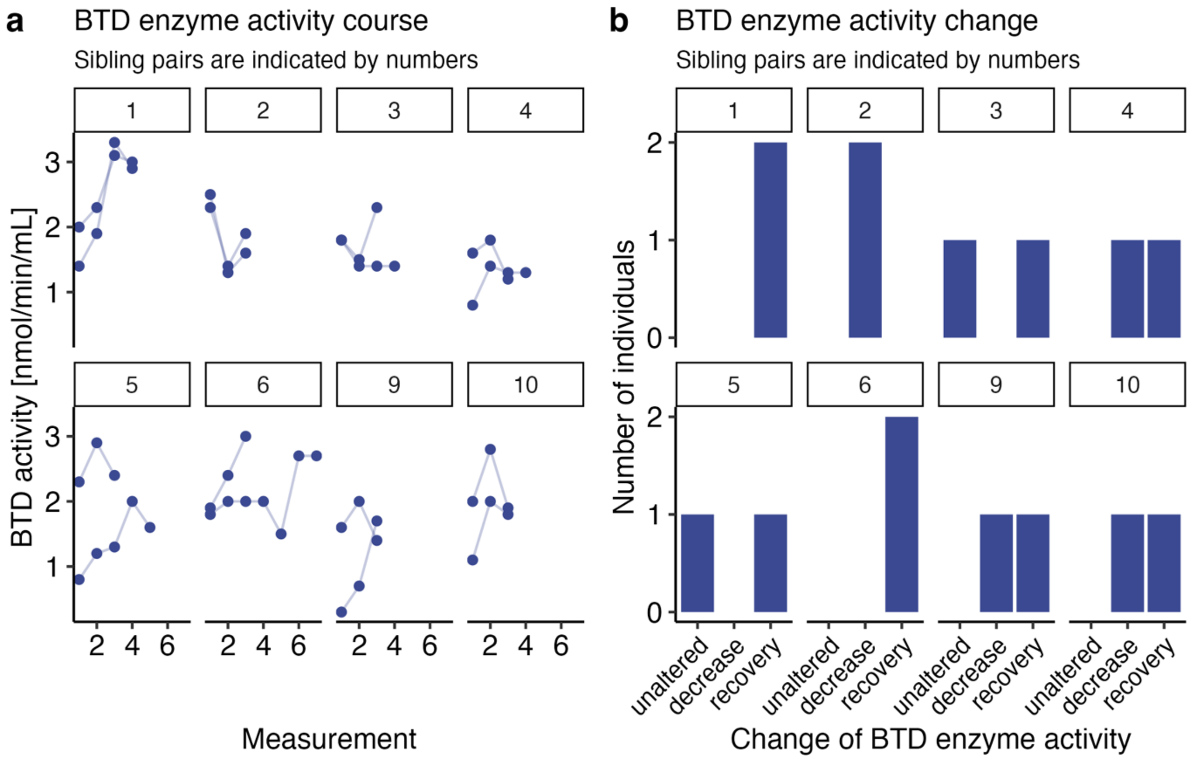

Supplementary Figure 4. Comparisons of sibling pairs. (**a**) Course of BTD enzyme activity at different measurement time points. (**b**) Category of BTD enzyme activity alteration with increasing age.
